# Supplementary material for: Emergent conformational and aggregation properties of synergistic antimicrobial peptide combinations
Source: Nanoscale. 2024 Oct 15;16(44):20657–69. doi: 10.1039/d4nr03043e (PMC11488577; doi:10.1039/d4nr03043e)
Supplement: NR-016-D4NR03043E-s001 [file NR-016-D4NR03043E-s001.pdf]

Supplementary Information (SI) for Nanoscale.
This journal is © The Royal Society of Chemistry 2024

Supplementary Information

Unsupervised learning elucidates the interplay between conformational flexibility and aggregation in synergistic antimicrobial peptides

Miruna Serian[1], A. James Mason[2], Christian D. Lorenz[1,3*]

[1]Biological Physics & Soft Matter Group, Department of Physics, King's College London, London, WC2R 2LS, United Kingdom.
[2]Institute of Pharmaceutical Science, School of Cancer & Pharmaceutical Science, King's College London, London SE1 9NH, United Kingdom.
[3]Department of Engineering, King's College London, London, WC2R 2LS, United Kingdom.

*Corresponding author(s). E-mail(s): chris.lorenz@kcl.ac.uk;

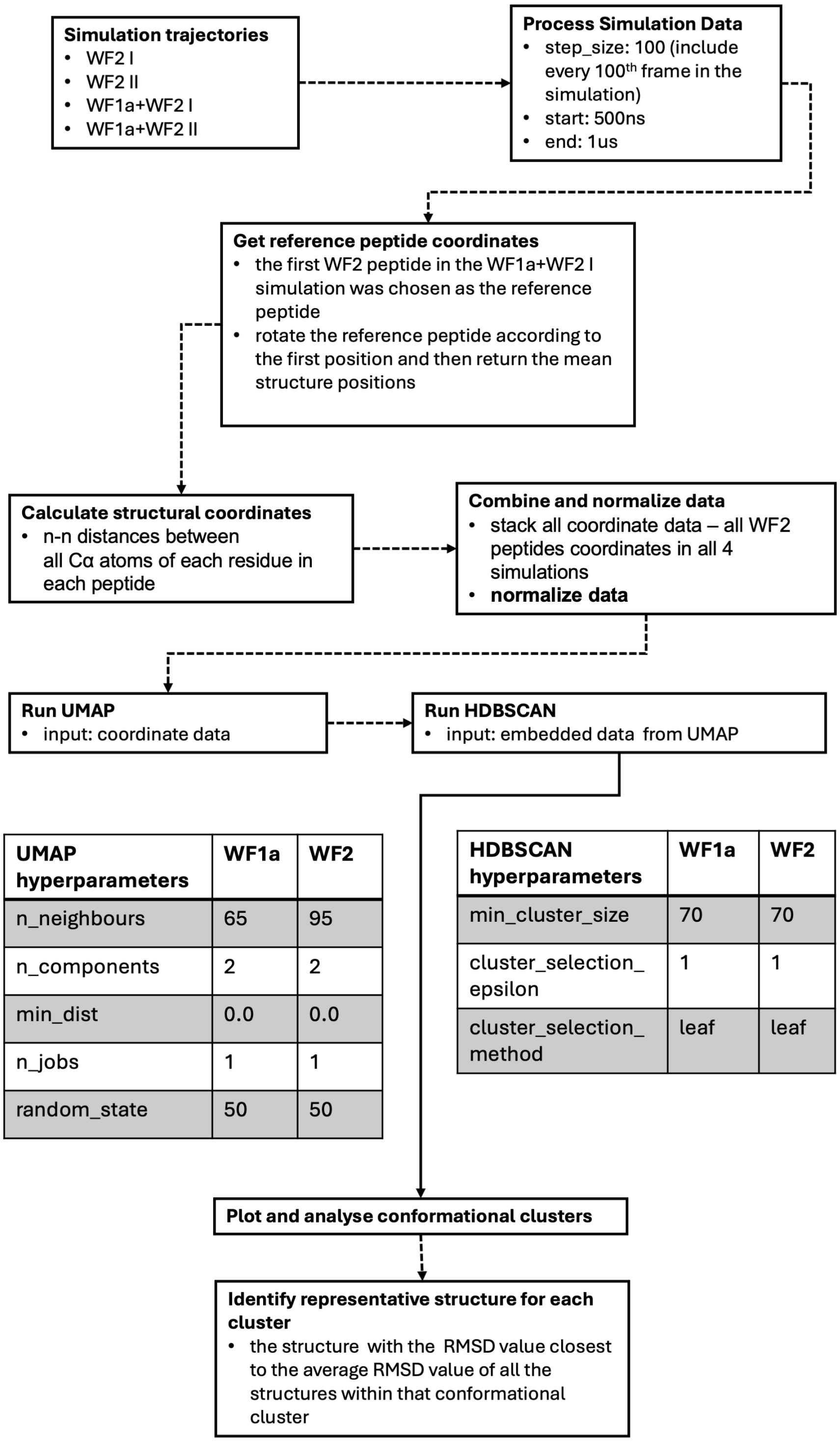

UMAP hyperparameters	WF1a	WF2
n_neighbours	65	95
n_components	2	2
min_dist	0.0	0.0
n_jobs	1	1
random_state	50	50

HDBSCAN hyperparameters	WF1a	WF2
min_cluster_size	70	70
cluster_selection_epsilon	1	1
cluster_selection_method	leaf	leaf

Fig. S1: Example of UMAP and HDBSCAN workflow for WF2 peptides. A similar workflow was applied for WF1a peptides.

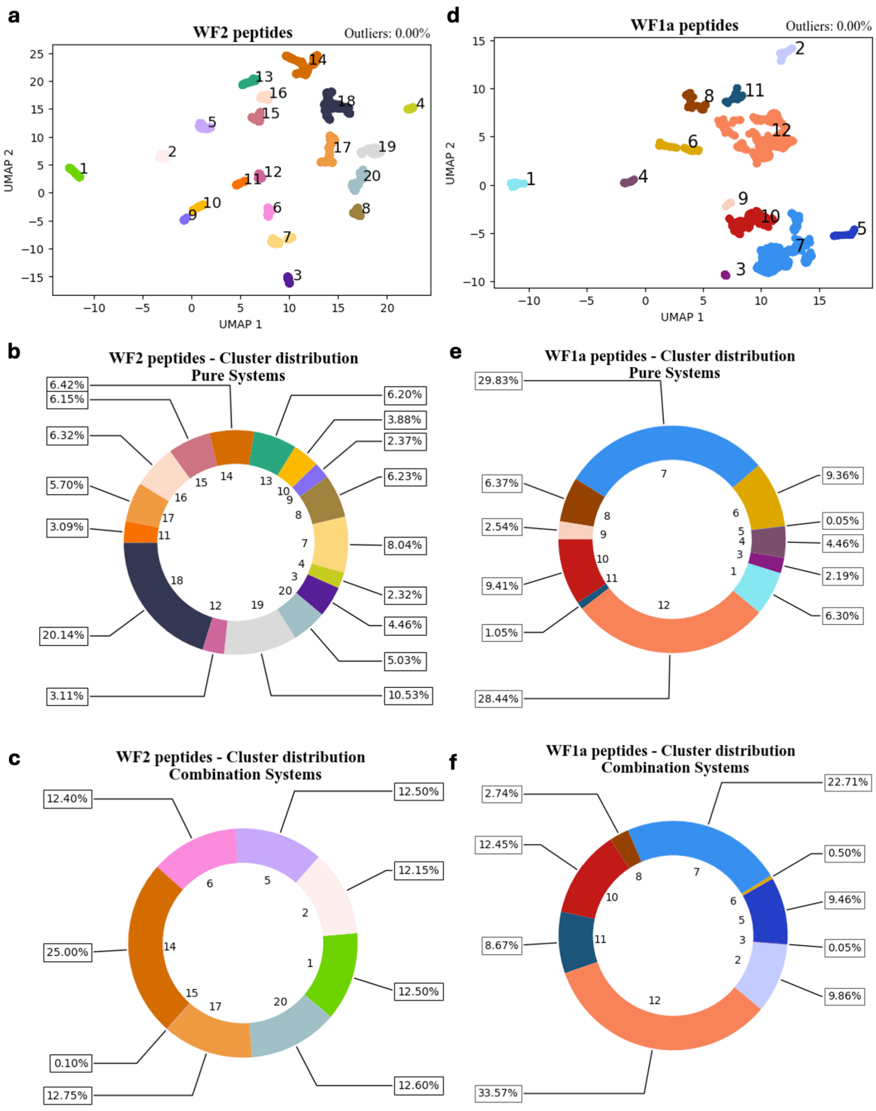

Fig. S2: Conformational clusters. UMAP projections in 2 dimensions for WF2 (a) and WF1a (d). The cluster distribution as percentages for WF2 - Pure systems (b), WF2 - Combination Systems (c) and WF1a - Pure Systems (e), WF1a - Combination Systems (f).

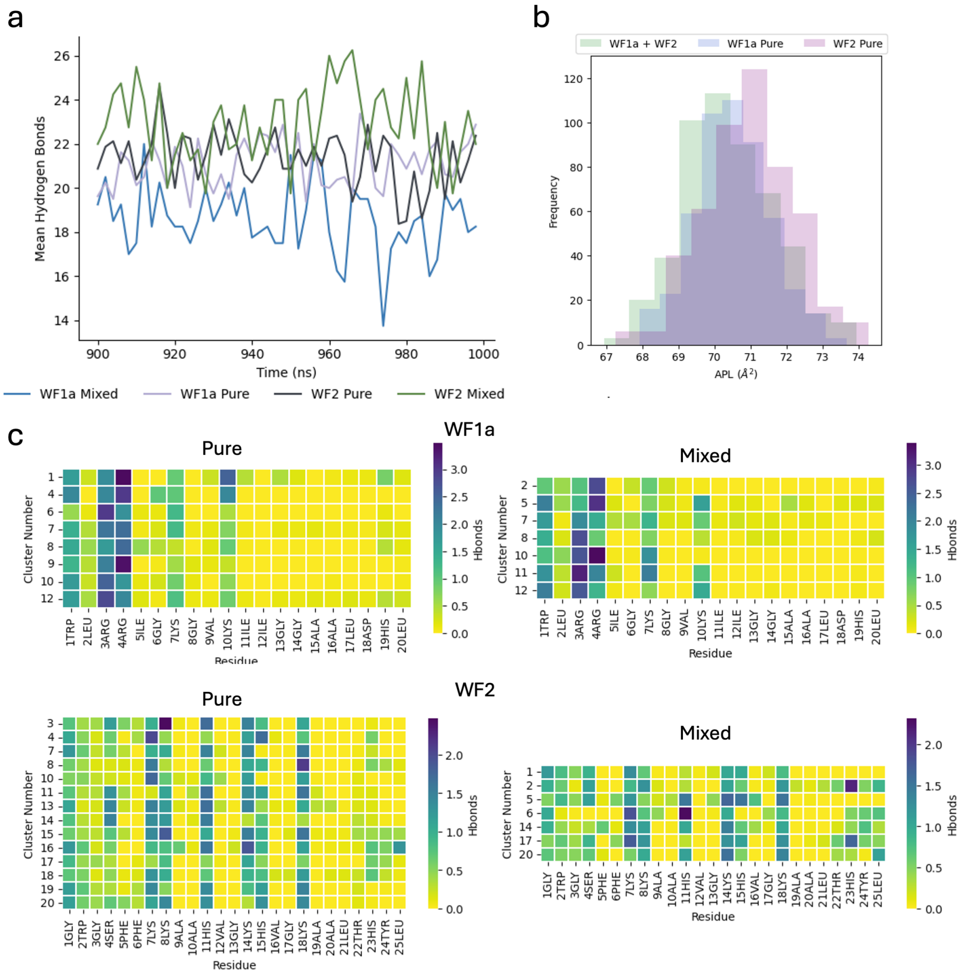

Fig. S3: Mean number of hydrogen bonds made with the membrane lipids per type of peptide and system during the last 100 ns of the simulations (a). Area per lipid (APL) distribution for each system during the last 50 ns of the simulations (b). Mean hydrogen bonds with membrane lipids per each residue and conformation (c).

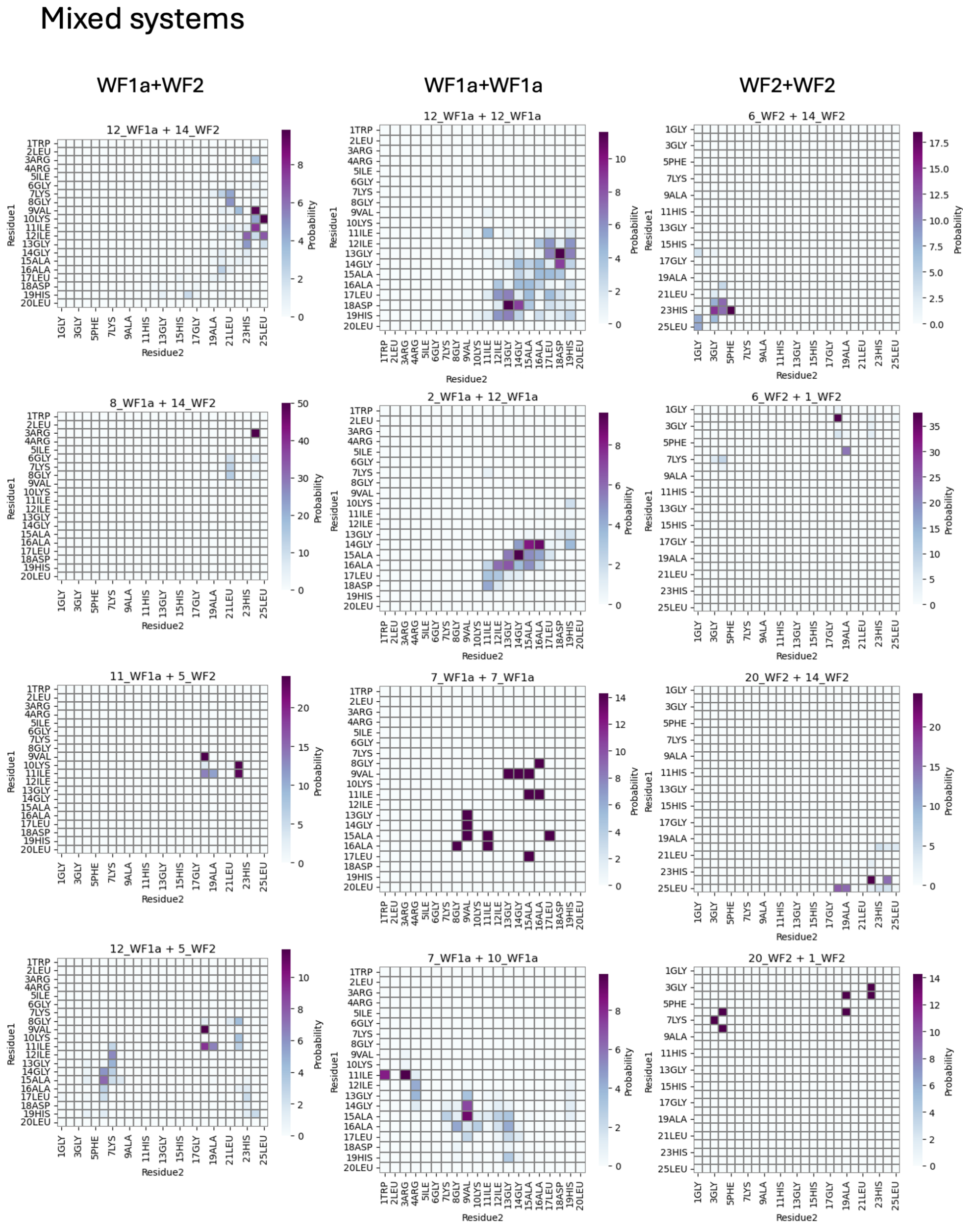

Fig. S4: Cluster-Cluster interaction probabilities in the mixed systems. Two residues are considered to be interacting if the distance between them is $\leq 6\mathring{A}$.

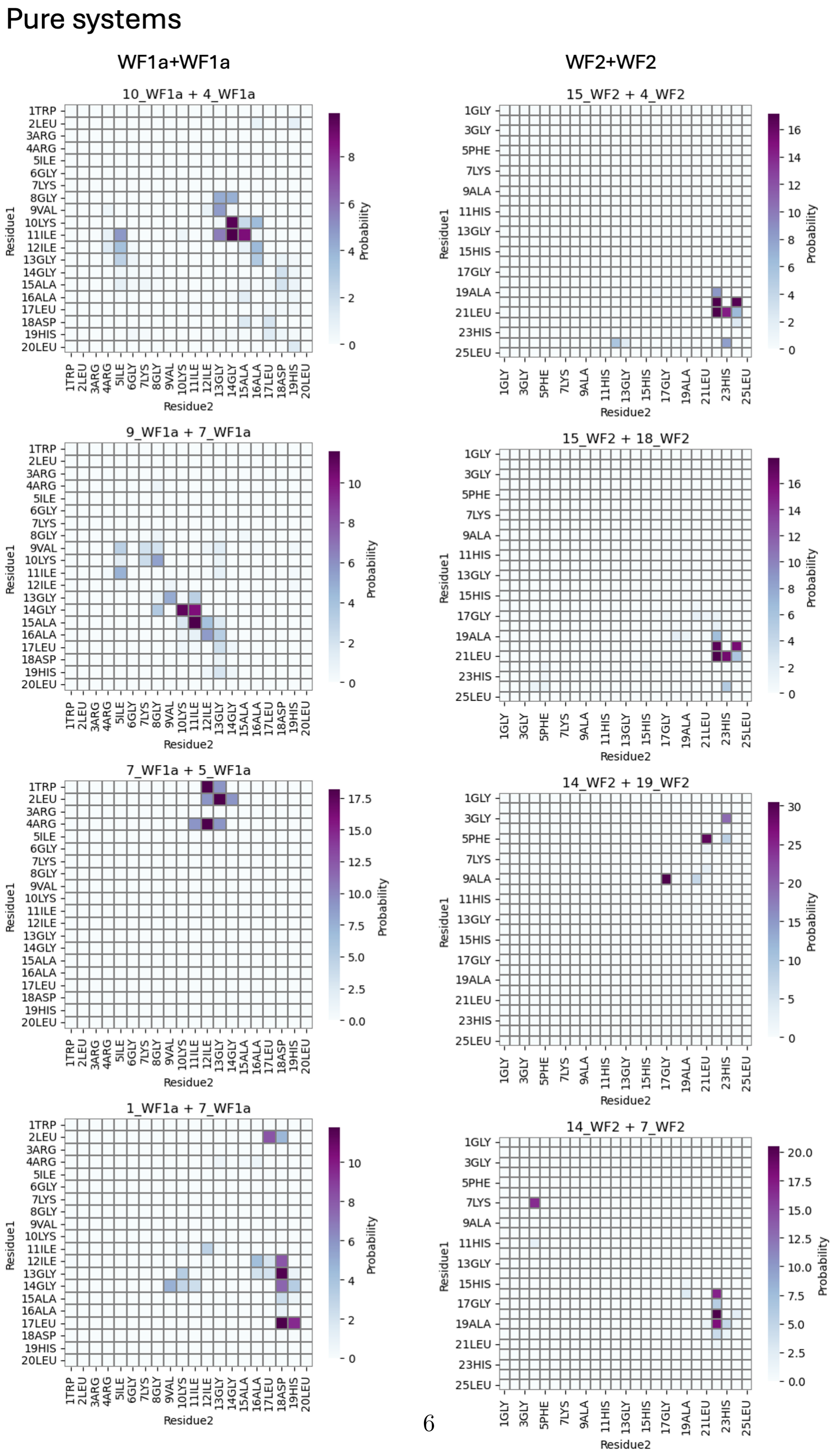

Fig. S5: Cluster-cluster interaction probabilities in the pure systems, grouped by the types of peptides involved. Two residues are considered to be interacting if the distance between them is $\leq 6\AA$.

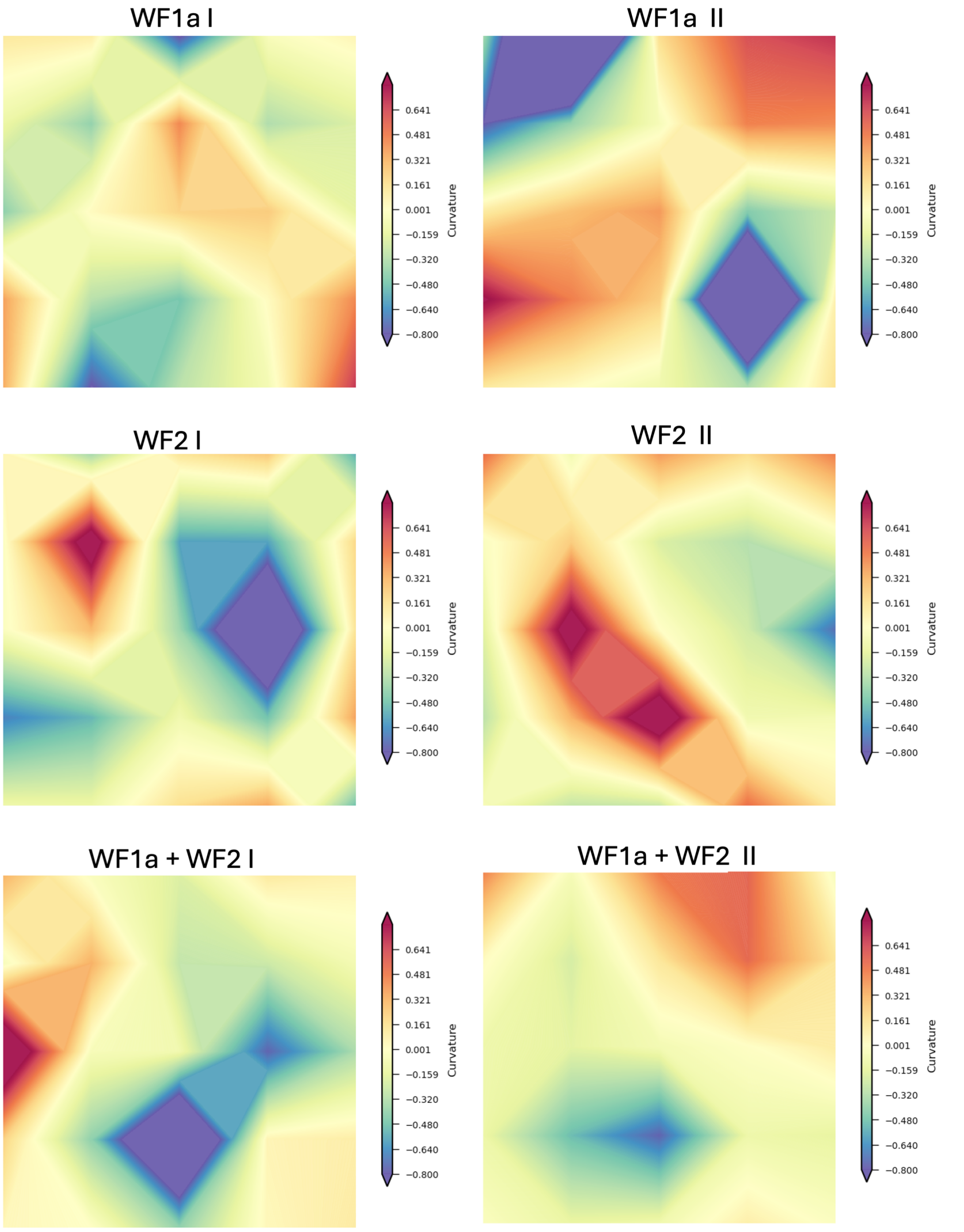

Fig. S6: The membrane curvature of the upper leaflet at 1 microsecond for each system and simulation. Negative values indicate a positive curvature while positive values indicate a negative curvature. The curvature data was generated using the LipidDyn Package [1].

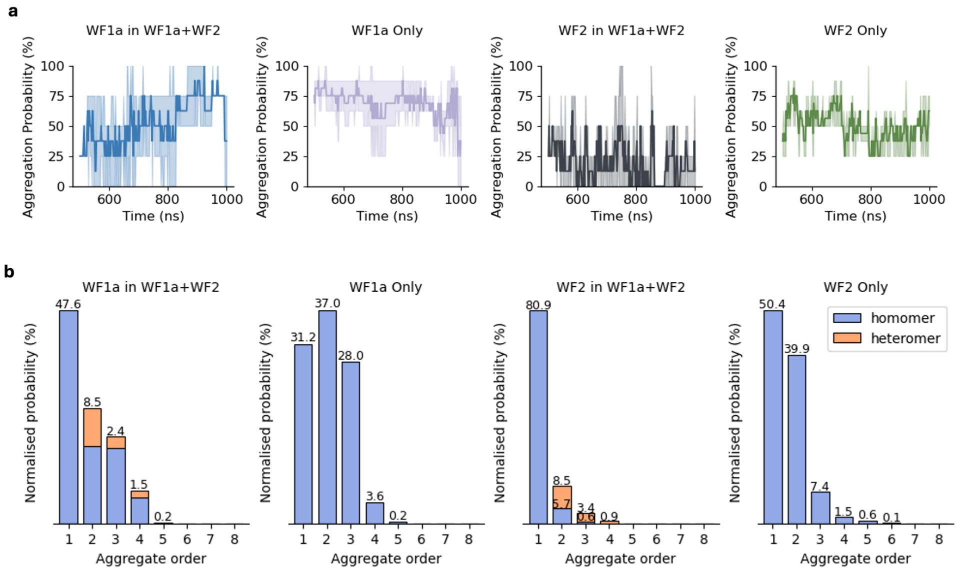

Fig. S7: Peptide aggregation probabilities for the peptides simulated in bulk water only. (a) The aggregation probability was calculated across all both types of systems and include both simulation runs. The values were normalised by the number of peptides of the same type in the system. The resulting values indicate the likelihood of a specific peptide type being present within an aggregate at any given time. (b) The distribution of aggregate order probabilities is depicted for each peptide type and system, illustrating the likelihood of being in an aggregate of a specific size.

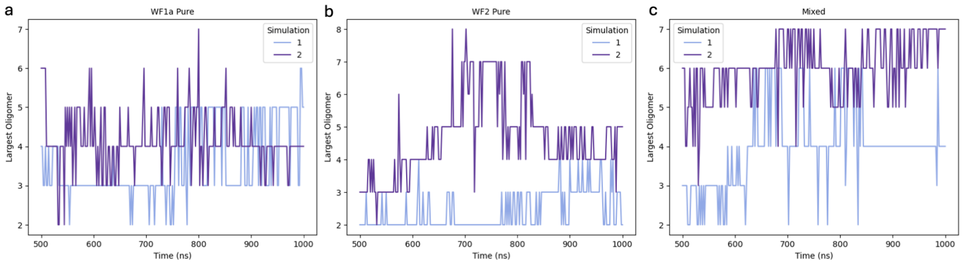

Fig. S8: Time evolution of the largest oligomer size in WF1a Pure (a), WF2 Pure (b) and Mixed (c) systems.

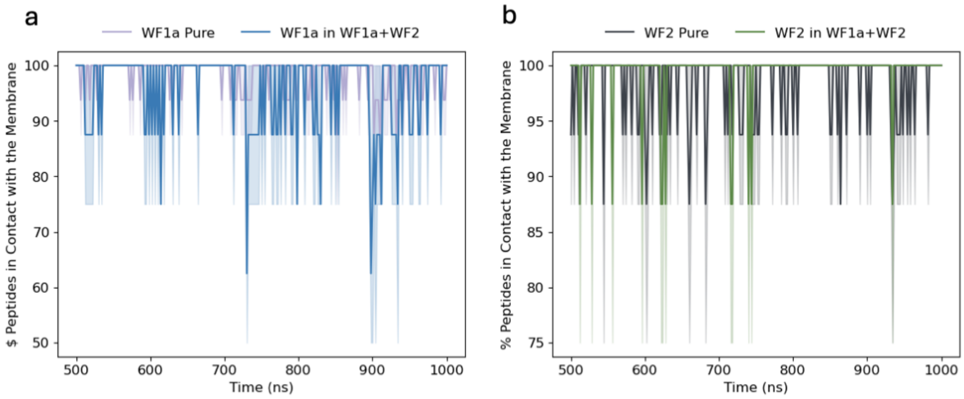

Fig. S9: The percentage of peptides in contact with the membrane for WF1a peptides (a) and WF2 peptides (b). A peptide is in contact with the membrane if the distance to the closest phosphorous atom in the membrane lipid head groups is ≤ 6 Å.

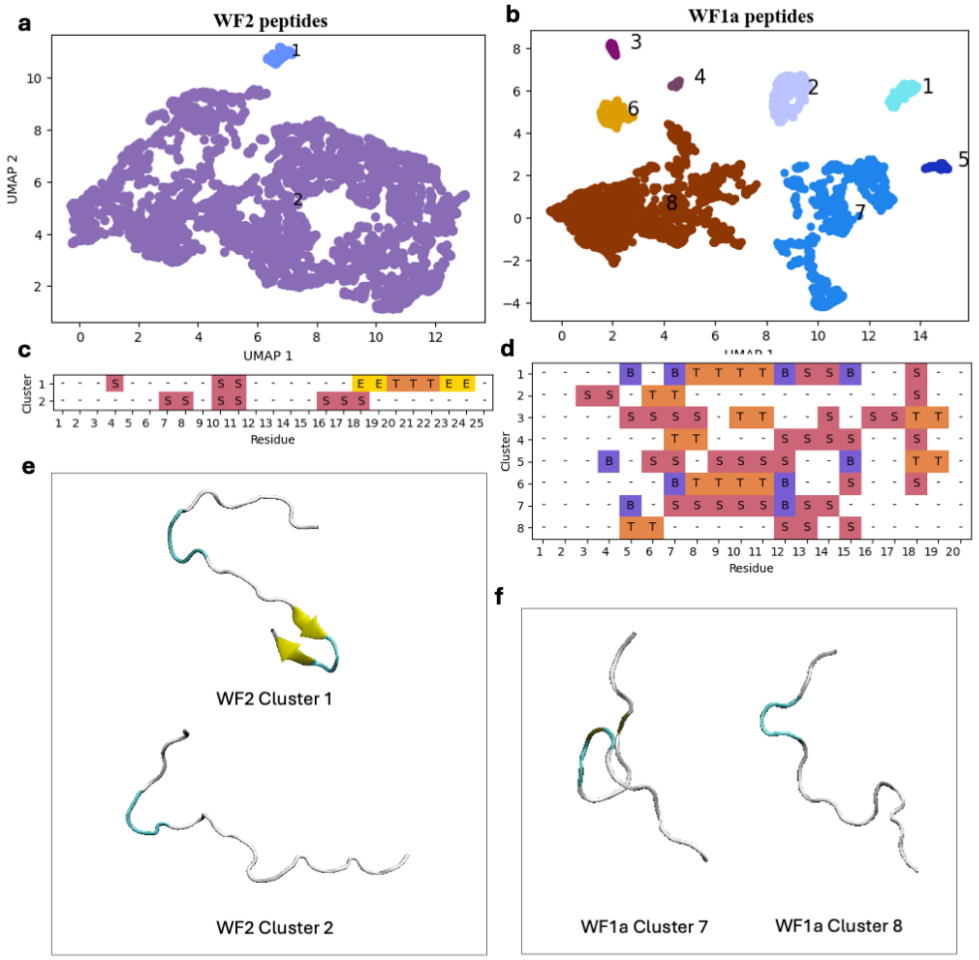

Fig. S10: Conformational clusters identified for the peptides simulated in water-only. The two-dimensional projection of the UMAP embedding for WF2 peptides is shown in (a), and for WF1a peptides in (b). The hyperparameters used form UMAP and HDBSCAN can be found in Table SI S1. The secondary structure assignment of the structures representative for each conformational cluster for WF2 peptides is shown in (c), and WF1a peptides in (d). Representative structures for the most common conformational clusters seen in WF2 peptides are displayed in (e), and WF1a peptides in (f).

Step	Hyperparameters	WF1a	WF2
UMAP	*n_neighbours*	65.0	35.0
	n_components	2.0	2.0
	min_dist	0.0	0.0
	random_state	40.0	40.0
	n_jobs	1.0	1.0
HDBSCAN	*min_cluster_size*	40.0	40.0
	cluster_selection_epsilon	1.0	1.0
	cluster_selection_method	leaf	leaf

Table S1: Hyperparameters used for UMAP and HDBSCAN for peptide-water systems

**Cluster**	**WF1a Combination**		**Pure**	
	**Mean**	**SD**	**Mean**	**SD**
**1**			1.69	0.65
**2**	1.77	0.66		
**5**	1.79	0.60	1.0	0.0
**6**	1.33	0.47	1.81	0.70
**7**	1.53	0.53	1.99	0.74
**8**			1.14	0.35
**9**			2.04	0.94
**10**			1.46	0.50
**12**	2.13	0.54	1.32	0.49

(a) WF1a peptides

**Cluster**	**WF2 Combination**		**Pure**	
	**Mean**	**SD**	**Mean**	**SD**
**1**	1.51	0.69		
**4**			1.00	0.00
**6**	1.82	0.38		
**7**			1.9	0.3
**11**			1.93	0.59
**12**			2.67	0.49
**14**	3.17	0.63	2.87	0.50
**15**			1.94	0.37
**16**			1.11	0.31
**17**			1.00	0.0
**18**			1.16	0.47
**19**			1.23	0.42
**20**	1.36	0.48		

(b) WF2 peptides

Table S2: Cluster centrality values for WF1a (a) and WF2 peptides (b). The values were computed using the degree_centrality() function of NetworkX library [2]

Cluster_pep1	Cluster_pep2	Probability %	System	pep1	pep2
12	14	5.12	WF1a_WF2	WF1a	WF2
11	5	3.27	WF1a_WF2	WF1a	WF2
12	5	2.19	WF1a_WF2	WF1a	WF2
8	14	1.67	WF1a_WF2	WF1a	WF2
2	12	8.04	WF1a_WF2	WF1a	WF1a
7	7	7.00	WF1a_WF2	WF1a	WF1a
7	10	4.38	WF1a_WF2	WF1a	WF1a
12	12	2.30	WF1a_WF2	WF1a	WF1a
12	14	5.12	WF1a_WF2	WF1a	WF2
11	5	3.27	WF1a_WF2	WF1a	WF2
12	5	2.19	WF1a_WF2	WF1a	WF2
8	14	1.67	WF1a_WF2	WF1a	WF2
10	4	9.55	WF1a_only	WF1a	WF1a
9	7	7.88	WF1a_only	WF1a	WF1a
7	5	5.50	WF1a_only	WF1a	WF1a
1	7	4.68	WF1a_only	WF1a	WF1a
6	14	4.04	WF1a_WF2	WF2	WF2
6	1	1.87	WF1a_WF2	WF2	WF2
20	14	1.20	WF1a_WF2	WF2	WF2
20	1	0.03	WF1a_WF2	WF2	WF2
15	4	5.84	WF2_only	WF2	WF2
15	18	3.59	WF2_only	WF2	WF2
14	19	3.03	WF2_only	WF2	WF2
14	7	2.82	WF2_only	WF2	WF2

Table S3: Cluster-cluster interactions. Only the top 5 interactions based on probability values for each type of interaction is displayed

	Peptide2	WF1a		
Peptide1	Cluster1	4	5	7
WF1a	1			17LEU:18ASP (11.8%) -vdW<br>13GLY:18ASP (11.6%) - vdW<br>17LEU:19HIS (9.5%) vdW<br>2LEU:17LEU (8.2%) - HI<br>12ILE:18ASP (8.1%) - vdW
	10	11ILE:14GLY (9.8%) -HI<br>10LYS:14GLY (9.5%) - vdW<br>11ILE:15ALA (8.7%) - HI<br>11ILE:13GLY (5.6%) - HI<br>11ILE:5ILE (4.9%) - HI		
	7		4ARG:12ILE (18.2%) - vdW<br>1TRP:12ILE (18.2%) -HI<br>2LEU:13GLY (18.2%) - HI<br>1TRP:13GLY (9.1%) HI<br>4ARG:11ILE (9.1%) -vdW	
	9			15ALA:11ILE (11.6%) - HI<br>14GLY:10LYS (11.1%) - vdW<br>14GLY:11ILE (10.2%) - HI<br>16ALA:12ILE (5.6%) - HI<br>10LYS:8GLY (5.3%)-vdW

Table S4: Top residue-residue contacts of type WF1a:WF1a in the pure WF1a systems

Peptide1	Peptide2<br>Cluster	WF1a<br>10	12	7
WF1a	12		18ASP:13GLY (8.7%) - vdW<br>17LEU:13GLY (6.5%)- HI<br>17LEU:12ILE (5.8%) -HI<br>19HIS:13GLY (5.8%) - vdW<br>18ASP:14GLY (5.8%)- vdW	
	2		15ALA:14GLY (9.6%)- HI<br>14GLY:16ALA (8.9%)- HI<br>14GLY:15ALA (8.2%) - HI<br>16ALA:13GLY (6.6%)- HI<br>16ALA:12ILE (6.0%) - HI	
	7	11ILE:3ARG (9.9%) - vdW<br>15ALA:9VAL (9.1%) - HI<br>11ILE:1TRP (8.3%) - HI<br>14GLY:9VAL (7.2%) - HI<br>16ALA:8GLY (4.4%)- HI		16ALA:11ILE (14.3%) - HI<br>16ALA:8GLY (14.3%)- HI<br>15ALA:9VAL - HI (14.3%)<br>15ALA:17LEU (14.3%) - HI<br>13GLY:9VAL (14.3%)- HI

Table S5: Top residue-residue contacts of type WF1a:WF1a in the mixed WF1a systems

	Peptide2	WF2	
Peptide1	Cluster1	1	14
WF2	20	6PHE:4SER (14.3%)- vdW<br>4SER:22THR (14.3%) - HB<br>7LYS:3GLY (14.3%) - vdW<br>4SER:19ALA (14.3%) -vDW<br>6PHE:19ALA - HI (14.3%)	24TYR:22THR (24.2%) -HB<br>25LEU:18LYS (16.2%) -vdW<br>25LEU:19ALA (15.2%) - HI<br>24TYR:24TYR (14.9%) - π-π<br>20ALA:23HIS (4.6%) - vdW
	6	2TRP:18LYS (37.6%) -vdW<br>6PHE:19ALA (22.3%) -HI<br>7LYS:4SER (9.0%) - electrostatic<br>4SER:18LYS (6.0%) - electrostatic<br>7LYS:3GLY (4.9%) -vdW	23HIS:5PHE (18.5%) - π-π<br>23HIS:3GLY (14.5%) -vdW<br>22THR:4SER (11.6%) - HB<br>23HIS:4SER (11.5%) - HB<br>25LEU:1GLY (8.1%) -vdW

Table S6: Top residue-residue contacts of type WF2:WF2 in the mixed WF2 systems

	Peptide2	WF2			
Peptide1	Cluster1	18	19	4	
WF2	14		9ALA:17GLY (30.5%) - HI<br>5PHE:21LEU (29.3%) -HI<br>3GLY:23HIS (19.1%) -vdW<br>5PHE:23HIS (8.1%) -π-π<br>9ALA:20ALA (7.0%) - HI		18LYS:22THR (20.5%) - HB<br>19ALA:22THR (18.1%) - vdW<br>16VAL:22THR (16.9%) - vdW<br>7LYS:4SER (16.5%) - electrostatic<br>17GLY:22THR (5.7%) -vdW
	15	21LEU:22THR (17.9%) -vdW<br>20ALA:22THR (17.3%) - vdW<br>21LEU:23HIS (16.3%) - vdW<br>20ALA:24TYR (16.1%) - vdW<br>19ALA:22THR (6.5%)-vdW		20ALA:22THR (17.1%) - vdW<br>21LEU:22THR (17.1%) - vdW<br>20ALA:24TYR (16.7%) - vdW<br>21LEU:23HIS (14.4%) - vdW<br>19ALA:22THR (8.5%) - vdW	

Table S7: Top residue-residue contacts of type WF2:WF2 in the mixed WF2 systems

References

[1] Scrima, S., Tiberti, M., Campo, A., Corcelle-Termeau, E., Judith, D., Foged, M.M., Clemmensen, K.K.B., Tooze, S.A., Jäättelä, M., Maeda, K., Lambrughi, M., Papaleo, E.: Unraveling membrane properties at the organelle-level with lipiddyn. Computational and Structural Biotechnology Journal **20**, 3604–3614 (2022) https://doi.org/10.1016/j.csbj.2022.06.054

[2] Hagberg, A.A., Schult, D.A., Swart, P.J.: Exploring network structure, dynamics, and function using networkx. In: Varoquaux, G., Vaught, T., Millman, J. (eds.) Proceedings of the 7th Python in Science Conference (SciPy2008), Pasadena, CA, USA, pp. 11–15 (2008)
